# Outcomes of pirtobrutinib for relapsed/refractory mantle cell lymphoma in compassionate use program in Europe

**DOI:** 10.1002/cam4.7289

**Published:** 2024-05-21

**Authors:** Enver Aydilek, Gerald Wulf, Friedrich Schwarz, Ulrike Bacher, Mathias Rummel, Olga Stiefel, Andrea Kerkhoff, Markus Maulhardt, Thomas Melchardt, Thomas Pabst, Georg Lenz, Evgenii Shumilov

**Affiliations:** ^1^ Department for Hematology and Medical Oncology University Medical Center Göttingen Göttingen Germany; ^2^ Campus Institute for Dynamics of Biological Networks, Georg August University Göttingen Germany; ^3^ Department of Hematology, Inselspital University Hospital Bern, University of Bern Bern Switzerland; ^4^ Department of Hematology, Clinic for Haematology and Medical Oncology Justus Liebig University Hospital Gießen Germany; ^5^ Division of Hematology With Stem Cell Transplantation, Hemostaseology and Medical Oncology, Department of Internal Medicine I Ordensklinikum Linz Linz Austria; ^6^ Department of Medicine A, Hematology, Oncology and Pneumology University Hospital Muenster Muenster Germany; ^7^ Department of Internal Medicine III with Haematology, Medical Oncology, Haemostaseology, Infectiology and Rheumatology Paracelsus Medical University Salzburg Austria; ^8^ Department of Medical Oncology Inselspital Bern University Hospital Bern Switzerland

**Keywords:** compassionate use program, mantle cell lymphoma, outcomes, pirtobrutinib

## Abstract

**Background:**

Mantle cell lymphoma (MCL) is a type of B‐cell lymphoma that is currently incurable. Pirtobrutinib shows promising response rates in heavily pretreated MCL patients according to the approval study, but the real‐world data are scarce.

**Methods:**

In this study, we retrospectively analyzed the efficacy and safety profile of pirtobrutinib in 10 relapsed/refractory MCL patients from compassionate use program (CUP).

**Results:**

On average, the patients underwent three lines of systemic therapy prior to pirtobrutinib and were predominantly BTKi exposed (9/10). The best overall response rate (BORR) was 67%. In a median follow‐up of 8.6 months, the mean duration of response (DOR), progression‐free survival (PFS), and overall survival (OS) were not reached. No new safety signals were documented.

**Conclusions:**

In summary, pirtobrutinib represented a safe and effective treatment option in a small real‐world population.

## Graphical abstract

Data describing outcomes of pirtobrutinib in patients with relapsed/refractory mantle‐cell lymphoma (r/r MCL) in clinical routine are lacking. Here, we present the findings of a real‐world data study including 10 patients with heavily pretreated r/r MCL (median 3 therapy lines prior) who underwent treatment with pirtobrutinib as part of the compassionate use patient program (CUP) in Germany, Austria, and Switzerland. Best overall response rate was 70%. One‐year PFS and OS were 56% and 70%, accordingly. No new safety signals were documented. The outcomes observed in our study were encouraging and similar to those reported in the pivotal study.

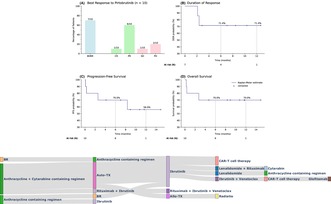

## 1 INTRODUCTION

Mantle cell lymphoma (MCL) accounts for 6%–8% of lymphomas arising from lymphocytes originating in the mantle zone.^1^ The translocation t(11;14)(q13;q32) is detectable in almost all MCL cases resulting in constitutive cyclin D1 overexpression that drives malignant B‐cell growth.^2^ Deregulation of B‐cell receptor (BCR) signaling pathway due to mutations or epigenetic events impacts regulatory proteins triggering cell proliferation.^3^ Bruton's tyrosine kinase (BTK) is a key component of the BCR signaling pathway.^4^ Discovering the role of BCR and BTK particularly in MCL pathobiology allowed to integrate the covalent, first generation BTK inhibitor (cBTKi), ibrutinib, in front‐ and second‐line therapies.^5^, ^6^

Up to 12/2022, anthracycline and cytarabine‐containing immunochemotherapy followed by autologous stem cell transplantation and anti‐CD20 antibody maintenance was standard‐of care (SOC) for front‐line treatment of younger MCL patients.^7^ This standard was successfully challenged by the phase III TRIANGLE study, with ibrutinib combined with above mentioned SOC (arm A + I) versus SOC only (arm A) and ibrutinib containing treatment without HDCT/ASCT (arm I). Three‐year failure‐free survival (FFS) was higher in arm A + I (88%) and arm I (86%) compared to arm A (72%).^5^ However, despite these advances MCL relapses still occur.

Also in relapsed/refractory (r/r) settings, cBTKis are efficacious in MCL. In the approval study, ibrutinib demonstrated a 68% overall response rate (ORR) and 47% 24‐months OS among r/r MCL patients with median 3 prior therapy lines.^8^, ^9^ Nevertheless, progression on ibrutinib is frequently detactable.^10^ One of the most common resistance mechanisms among non‐responders is a mutation in the *BTK481* cysteine residue which blocks covalent binding of irreversible BTK inhibitors, e.g. ibrutinib, or second cBTKis generation acalabrutinib and zanubrutinib.^11^, ^12^

Recently, non‐covalent BTKi pirtobrutinib was shown to overcome resistance to covalent BTKi. While ibrutinib and the second BTKi generation covalently bind to BTK, pirtobrutinib forms a non‐covalent bond with the latter enabling a selective and reversible inhibition of BTK activity even after failure of cBTKis.^13^ In the approval, phase I/II BRUIN trial, pirtobrutinib induced encouraging and sustained ORR of 57.8% (52/90) including 20% (18/90) complete responses (CR) among r/r MCL patients prior cBTKi. Furthermore, pirtobrutinib demonstrated good tolerability and a favorable safety profile. Correspondingly, FDA and EMA granted accelerated approval and conditional marketing authorization of pirtobrutinib in r/r MCL after at least two and one prior therapy lines, including cBTKi, in 01/2023 and 10/2023, respectively.^14^, ^15^

Collecting data from patients who have received pirtobrutinib outside of a controlled clinical trial setting can improve insights into clinical applicability, treatment duration, effectiveness, and safety in patients with r/r MCL. To this end, we performed a retrospective, multicenter, real‐world data study including 10 patients with r/r MCL who received pirtobrutinib within a compassionate use patient program (CUP) in Germany, Austria, and Switzerland.

## 2 MATERIALS AND METHODS

This retrospective study enrolled 10 consecutive patients with r/r MCL who underwent pirtobrutinib therapy between 08/2022 and 11/2023 within CUP prior to EMA approval. Patients were deemed eligible for the CUP in the case of failure of all available treatment options and adequate organ function. BTKi naive patients were considered for CUP on the same conditions as those being BTKi pre‐treated. The outcomes of CUP patients were compared with the results of previously reported approval study (BRUIN Trial).^16^ The permission to publish the CUP data was obtained in advance from the provider of pirtobrutinib, firma Lilly.

All patients provided informed consent before treatment. Data from different centers were anonymized before being pooled for joint analysis to ensure patient confidentiality and comply with data protection regulations. Study protocols and procedures adhered to pertinent guidelines, including Helsinki Declaration, in addition to local regulations. The study received approval from the respective local ethics committees (Göttingen №: 15/6/23). Detailed description of patient stratification, response assessment, adverse event grading, endpoints and statistical analysis is presented in Supplemental Material “Methods and Statistics”.

## 3 RESULTS

### 3.1 Patient characteristics

Patient characteristics and information on previous therapies are summarized in Table S1. The median age at pirtobrutinib initiation was 72 years, the majority of patients being male (6/10). Seven patients presented with high‐risk disease according to the MCL International Prognostic Index score (MIPI) and combinate MCL International Prognostic Index score (MIPIc). Figure S1 presents an overview on the therapies prior to pirtobrutinib.

In median, patients had undergone three therapy lines (range, 2–5) prior to pirtobrutinib and all had a history of immunochemotherapy (10/10). Nine of 10 patients had received ibrutinib, of them two subsequently combined with BCL‐2 inhibitor venetoclax +/− rituximab following progression under BTKi alone. The reason for BTKi discontinuation was either progressive disease (PD) (7/9) or intolerability (2/9). The cellular therapies prior to pirtobrutinib encompassed stem cell transplantation in 7/10 cases, of them 6 autologous and 1 allogeneic SCT, and CD19‐CAR‐T cell therapy (3/10). Two patients received lenalidomide, one patient glofitamab. At the time of pirtobrutinib initiation, all patients presented with PD (10/10).

### 3.2 Outcomes of treatment with pirtobrutinib

**FIGURE 1:**
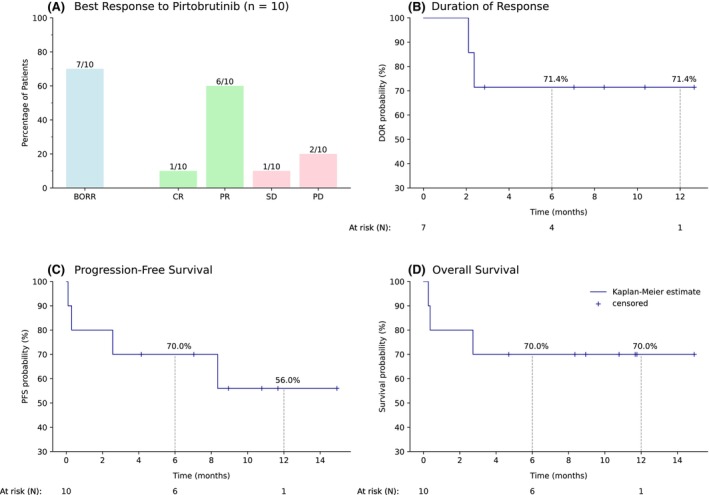
Outcome of treatment from the 10 patients with r/r MCL and pirtobrutinib. (A) Best overall response rates of the patients treated with pirtobrutinib. (B) Duration of response (DOR). (C) Progression‐free survival (PFS). (D) Overall survival (OS). BORR, best overall response rate; CR, complete response; PR, partial response; SD, stable disease; PD, progressive disease. For DOR analysis, only patients showing a response were included. All patients were included for PFS and OS analysis. The survival percentages for 6 and 12 months are given.

The outcomes of pirtobrutinib are presented in Table 1 and Figure 1. The median follow‐up (FU) from pirtobrutinib initiation was 8.6 months (range, 0.3–14.9 months). The best overall response rate (BORR) was 70% (7/10): one patient achieved CR, while six exhibited partial response (PR) (Figure 1A). The median time to best response was 70 days (range, 14–167). The remaining patients (3/10) experienced either SD (1/10) or PD (2/10). Of BTKi pretreated patients (9/10), a response was detectable in 6 out of 9. One patient with PR underwent subsequently consolidative CAR‐T cell therapy following 3 months of pirtobrutinib.

**TABLE 1:** Outcomes of pirtobrutinib treatment among patients of the study and approval trial.

Response	CUP, all patients (*n* = 10)	cBTKi pretreated MCL (*n* = 90)	cBTKi‐naive MCL (*n* = 14)
Overall response rate, % (95% CI)	70 (34.8 to 93.3)	57.8 (46.9 to 68.1)	85.7 (57.2 to 98.2)
Best overall response, No. (%)			
Complete response	1 (10)	18 (20.0)	5 (35.7)
Partial response	6 (60)	34 (37.8)	7 (50)
Stable disease	1 (10)	14 (15.6)	—
Progressive disease	2 (20)	15 (16.7)	1 (7.1)
Not evaluable	—	9 (10.0)	1 (7.1)
DOR			
Patients with a response, No. (%)	7 (70)	52 (57.8)	12 (85.7)
Patients with censored data, No. (%)	5 (71.4)	33 (63.5)	12 (100)
DOR, months, median (95% CI)	NR (2.37 to NR)	21.6 (7.5 to NR)	NR (NR to NR)
Median follow‐up, months	7.0	11.9	7.1
PFS			
Patients with censored data, No. (%)	7 (70)	45 (50.0)	13 (92.9)
PFS, months, median (95% CI)	NR (2.56 to NR)	7.4 (5.3 to 12.5)	NR (NR to NR)
Median follow‐up, months	7.7	9.2	8.6
OS			
Patients with censored data, No (%)	7 (70)	60 (66.7)	13 (92.9)
OS, months, median (95% CI)	NR (2.73 to NR)	NR (14.8 to NR)	NR (NR to NR)
Median follow‐up, months	8.6	16.6	9.4
Estimated 6 and 12 months DOR and PFS			
Estimated DOR, 6 months	71.4%	73.6%	
Estimated DOR, 12 months	71.4%	57.1%	
Estimated PFS, 6 months	70%	51.9%	
Estimated PFS, 12 months	56%	40%	

*Note*: One patient who did not undergo post‐baseline disease assessment is not included in the evaluation. The KM estimators' confidence intervals (CIs) are calculated using the exact binomial proportion CIs.

Abbreviations: cBTKi, covalent bruton tyrosine kinase inhibitor; CUP, compassionate use program; DOR, duration of response; MCL, mantle cell lymphoma; NR, not reached; OS, overall survival; PFS, progression‐free survival.

Within the responding patients (*n* = 7), the median duration of response (DOR) was not reached (95% CI: 2.37‐NR months) (Figure 1B) while 6‐ and 12‐month outcomes for DOR were 71.4%. Median PFS and OS were not reached: 95% CI of 2.56‐NR months and 2.37‐NR months, respectively. The estimated 6‐ and 12‐month outcomes for PFS and OS were 70% and 56% and 70% and 70% (Figure 1C,D). Given our follow‐up period, the mean observed DOR was 9.7 months (95%CI: 5.9‐NR, 12.7 months observation), and PFS 9.8 (95%CI: 5.9–13.7) and OS 10.8 months (95%CI: 6.8–14.7) for 14.9 months observation (Table S2).

### 3.3 Adverse Events

Non‐ and hematologic toxicities are listed in Table S3. Hematologic toxicity was mild and infrequent, no Grade 3 and 4 events were observed. The most common non‐hematologic toxicity (all CTCAE grade 1) was hemorrhage (2/10), followed by elevated liver enzymes, diarrhea, stomach pain, and dysgeusia (1/10 for each). No other AEs were documented.

## 4 DISCUSSION

Encouraging data regarding the efficacy and toxicity of pirtobrutinib have been generated for patients with r/r MCL in the approval study. In our—albeit small—retrospective, multicentric, international study we analyzed outcomes and safety of pirtobrutinib in r/r MCL patients undergoing treatment in real‐world settings within the Global Compassionate Use Program in Germany, Austria, and Switzerland.

Our patients and those in the approval study exhibited similar baseline clinical characteristics concerning gender and age, while blastoid histology and high‐risk MIPI/MIPIc score were more frequent in our analysis: 2/10 (20%) and 7/10 (70%) versus 8/90 (9%) and 20/90 (22%) respectively. Patients of this study as well as BTKi exposed patients from the pivotal study presented with a median of 3 prior therapy lines. Three patients received CAR‐T cell therapy preceding pirtobrutinib, compared to 4/90 in the pivotal study.

Response evaluations were at the physician's discretion, as is common in real‐world settings. Seven out of ten patient showed a response in our cohort, compared to 16/23 (69%) in the pivotal trial when consider only patients treated with 200 mg pirtobrutinib daily. Of note, the comparison of the patients who responded was slightly in favor of our cohort (6/9) (67%) versus 52/90 (58%) when compared with all BTKi exposed patients treated with different doses of pirtobrutinib (ranging from 25 to 300 mg daily) in the approval study.

The median follow‐up in the current analysis was shorter than in the pivotal trial: 8.6 versus 16.6 months. DOR and survival outcomes were comparable with the approval study. Particularly, the estimated 6‐month DOR and PFS were 71.4% and 70% compared to 73.6% and 51.9% in the pivotal trial while 12‐month DOR and PFS were documented with 71.4% and 56% versus 57.1% and 40%, accordingly.

The hematological and non‐hematological toxicity profile in our analysis provided no new safety signals. Particularly, no AEs grade >2 including atrial fibrillation of any grade were documented in our highly pretreated patient cohort. Thus, our data support that the favorable toxicity profile of pirtobrutinib as reported in the approval study.

Limitations of our study are its retrospective design, as well as the limited sample size and follow‐up time, thereby reducing the capability of long‐term conclusions about safety and efficacy of pirtobrutinib. Additionally, an efficacy of pirtobrutinib depending on BTK C481 and/or TP53 mutation status was not assessed due to the lacking data on the latter.

As far as we are aware, our study represents the first real‐world investigation of the effectiveness and tolerability of pirtobrutinib in the treatment of patients with r/r MCL. The response rates in our study were encouraging and similar to those in the pivotal study. Furthermore, there were no new safety signals concerning the toxicity profile.

To thoroughly assess the effectiveness, safety, and potential of pirtobrutinib for r/r MCL in the real‐world population, further research is necessary. Extending the duration of follow‐up in our study is crucial to improve understanding of the durability of pirtobrutinib treatment as well as increasing the sample size.

In summary, pirtobrutinib represents a safe and effective treatment option in a small real‐world population. Further research is warranted to generate more data on the use of pirtobrutinib in the clinical routine in patients with r/r MCL.

## AUTHOR CONTRIBUTIONS

**Enver Aydilek:** Conceptualization (lead); data curation (lead); formal analysis (equal); investigation (equal); methodology (equal); project administration (equal); resources (equal); software (equal); validation (equal); visualization (equal); writing – original draft (lead); writing – review and editing (equal). **Gerald Wulf:** Conceptualization (equal); data curation (equal); supervision (equal); validation (equal); writing – review and editing (equal). **Friedrich Schwarz:** Data curation (equal); formal analysis (equal); investigation (equal); methodology (equal); validation (equal); visualization (equal); writing – original draft (equal). **Ulrike Bacher:** Data curation (equal); validation (equal); writing – original draft (equal); writing – review and editing (equal). **Mathias Rummel:** Data curation (equal); validation (equal). **Olga Stiefel:** Data curation (equal); validation (equal). **Andrea Kerkhoff:** Data curation (equal); validation (equal). **Markus Maulhardt:** Data curation (equal); validation (equal). **Thomas Melchardt:** Data curation (equal); validation (equal). **Thomas Pabst:** Data curation (equal); validation (equal). **Georg Lenz:** Conceptualization (equal); data curation (equal); supervision (equal); validation (equal); writing – review and editing (equal). **Evgenii Shumilov:** Conceptualization (equal); data curation (equal); formal analysis (equal); investigation (equal); methodology (equal); project administration (equal); resources (equal); software (equal); supervision (lead); validation (equal); visualization (equal); writing – original draft (equal); writing – review and editing (equal).

## FUNDING INFORMATION

This research received no external funding.

## CONFLICT OF INTEREST STATEMENT

G.L. received research grants not related to this manuscript from AGIOS, AQUINOX, AstraZeneca, Bayer, Celgene, Gilead, Janssen, Morphosys, Novartis, Roche, and Verastem. G.L. received honoraria from ADC Therapeutics, Abbvie, Amgen, AstraZeneca, Bayer, BMS, Celgene, Constellation, Genase, Genmab, Gilead, Hexal/Sandoz, Immagene, Incyte, Janssen, Karyopharm, Lilly, Miltenyi, Morphosys, MSD, NanoString, Novartis, PentixaPharm, Roche, and Sobi.

## ETHICS STATEMENT

The study was conducted according to the guidelines of the Declaration of Helsinki, and approved by the Ethics Committee of the University Medical Center Göttingen No 15/6/23.

## INFORMED CONSENT STATEMENT

All patients provided informed consent before treatment initiation.

## CODE AVAILABILITY

All scripts used for the analysis are available upon reasonable request.

## Supporting information


Data S1:

Figure S1:

Table S1.

## Data Availability

The datasets generated and/or analyzed during the current study are not publicly available due to privacy and ethical restrictions but are available from the corresponding author on reasonable request.
