# Supplementary material for: Outcomes of pirtobrutinib for relapsed/refractory mantle cell lymphoma in compassionate use program in Europe
Source: Cancer Med. 2024 May 21;13(10):e7289. doi: 10.1002/cam4.7289 (PMC11106640; doi:10.1002/cam4.7289)
Supplement: Supplementary file 1 — Data S1: [file CAM4-13-e7289-s002.docx]

**Supplemental material: methods and statistics.**

**Patient Stratification, Response Assessment and Adverse Event Grading**

The recommended dosage of pirtobrutinib was 200 mg orally once daily until disease progression, unacceptable toxicity or entering subsequent treatment if used as bridging therapy. Response to pirtobrutinib therapy was categorized as follows: complete response (CR), partial response (PR), stable disease (SD), and progressive disease (PD). The best overall response rate (BORR) was defined as the proportion of patients achieving CR or PR as their best response. Response assessment was based on laboratory and/or radiologic criteria using sonographic, computed tomography (CT) and/or positron emission tomography (PET-CT) performed after initiation of pirtobrutinib therapy.

Adverse events occurring during pirtobrutinib therapy were assessed in accordance with the recommendations outlined in the Common Terminology Criteria for Adverse Events (CTCAE), Version 5.0(14). The data cutoff for the evaluation of outcomes was set on November 21, 2023.

**Endpoints and statistical analysis**

The primary endpoints of this study were best overall response rate (BORR), progression-free survival (PFS), and overall survival (OS) in patients who received pirtobrutinib treatment. Secondary endpoints encompassed duration of response (DOR) and the assessment of non-hematologic and hematologic toxicities following pirtobrutinib initiation.

Descriptive statistical analyses of the patient cohort (demographics and medical history) and efficacy and safety analyses of pirtobrutinib treatment were performed. BORR, DOR, PFS, and OS measured efficacy. Kaplan-Meier (KM) estimators were used to visualize DOR, PFS and OS probabilities. Right-censoring was used when an event did not occur within the observation period. Disease progression, or death was defined as an event for DOR. As the median survival did not fall below 50% in our observation period, we report additionally the restricted mean survival times (RMSTs) using the respective maximum follow-up as the upper limit for DOR, PFS, and OS. In the DOR analysis, only patients showing a response were included. The KM estimators' confidence intervals (CIs) are calculated using the exact binomial proportion CIs.

We contextualized our analysis by comparing our real-world data to the BRUIN trial.

All analyses, including statistical calculations and visualizations, were performed using Python version 3.11 with custom in-house scripts.
