# Supplementary figures and images for: Outcomes of pirtobrutinib for relapsed/refractory mantle cell lymphoma in compassionate use program in Europe
Source: Cancer Med. 2024 May 21;13(10):e7289. doi: 10.1002/cam4.7289 (PMC11106640; doi:10.1002/cam4.7289)

Supplemental Figure 1: The comprehensive overview of therapies prior to pirtobrutinib

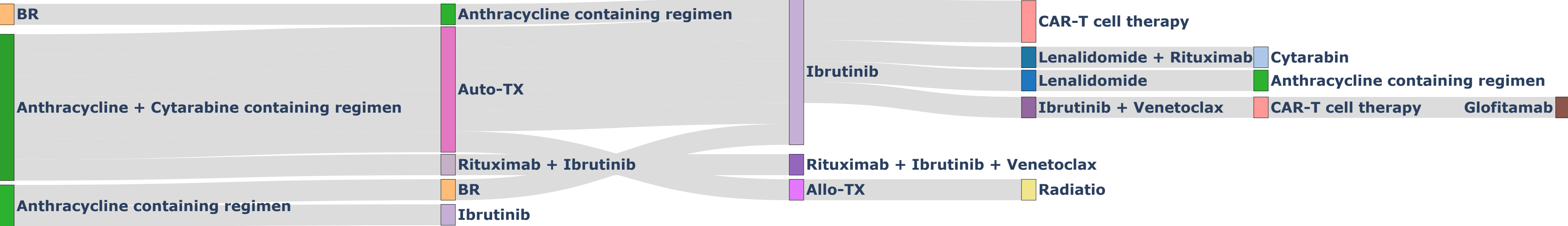

Supplement: Supplementary file 2 — Figure S1: [file CAM4-13-e7289-s001.pdf]
