# Supplementary material for: Outcomes of pirtobrutinib for relapsed/refractory mantle cell lymphoma in compassionate use program in Europe
Source: Cancer Med. 2024 May 21;13(10):e7289. doi: 10.1002/cam4.7289 (PMC11106640; doi:10.1002/cam4.7289)
Supplement: Supplementary file 3 — Table S1. [file CAM4-13-e7289-s003.docx]

**Supplemental Table 1**. Baseline characteristics of the patients of the study and approval (BRUIN) trial.

| Parameters | CUP pts (n=10) | cBTKi pretreated pts, BRUIN trial (n=90) | cBTKi naive pts,  BRUIN trial (n = 14) |
| --- | --- | --- | --- |
| **Age, years, median (range)** | 72.5 (56-77) | 70 (46-87) | 67 (60-86) |
| **Sex, No. (%)** | | |  |
| Female | 4 (40) | 18 (20.0) | 4 (28.6) |
| Male | 6 (60) | 72 (80.0) | 10 (71.4) |
| **Histology, No. (%)** | | |  |
| Classic | 6 (60) | 70 (77.8) | 11 (78.6) |
| Pleomorphic | - | 12 (13.3) | 2 (14.3) |
| Blastoid | 2 (20) | 8 (8.9) | 1 (7.1) |
| Unkown | 2 (20) | - | - |
| **ECOG PS, No. (%)** | | |  |
| 0 | 9 (90) | 61 (67.8) | 5 (35.7) |
| 1 | 1 (10) | 28 (31.1) | 8 (57.1) |
| 2 | - | 1 (1.1) | 1 (7.1) |
| **sMIPI/MIPI/MIPIc score, No. (%)** | | |  |
| Low risk (0-3) | - | 20 (22.2) | 3 (21.4) |
| Intermediate risk (4-5) | - | 50 (55.6) | 5 (35.7) |
| High risk (6-11) | 7 (70) | 20 (22.2) | 6 (42.9) |
| Unknown | 3 (30) | - | - |
| **Tumor bulk (cm), No. (%)** | | |  |
| <5 | 5 (50) | 66 (73.3) | 9 (64.3) |
| ≥5 | 4 (40) | 24 (26.7) | 5 (35.7) |
| ≥10 | - | 3 (3.3) | 2 (14.3) |
| Unknown | 1 (10) | - | - |
| **Extranodal disease, No. (%)** | | |  |
| Yes | 5 (50) | 35 (38.9) | 6 (42.9) |
| No | 4 (40) | 55 (61.1) | 8 (57.1) |
| Unkown | 1 (10) | - | - |
| **Bone marrow involvement, No. (%)** | | |  |
| Yes | 9 (90) | 46 (51.1) | 4 (28.6) |
| No | 1 (10) | 44 (48.9) | 10 (71.4) |
| **Prior lines of systemic therapy, No., median (range)** | 3 (2-5) | 3 (1-8) | 2 (1-3) |
| **Prior therapy, No. (%)** | | |  |
| Anti-CD20 antibody | 10 (100) | 86 (95.6) | 14 (100.0) |
| Chemotherapy | 10 (100) | 79 (87.8) | 14 (100.0) |
| cBTK inhibitor | 9 (90) | 90 (100.0) | - |
| Stem-cell transplant | 7 (70) | 19 (21.1) | 7 (50.0) |
| Autologous | 6 (60) | 17 (18.9) | 7 (50.0) |
| Allogeneic | 1 (10) | 4 (4.4) | 0 (0) |
| CAR-T cell therapy | 3 (30) | 4 (4.4) | 0 (0) |
| BCL-2 inhibitor | 2 (20) | 14 (15.6) | 0 (0) |
| Immunomodulator | 2 (20) | 19 (21.1) | 1 (7.1) |
| PI3K inhibitor | - | 3 (3.3) | 1 (7.1) |
| CD3-CD20 bispecific antibody | 1 (10) | - | - |
| **Reason discontinued any previous cBTK inhibitor,**^a,b^ **No. (%)** | | |  |
| Progressive disease | 7 (77.8) | 74 (82.2) | - |
| Toxicity/other^c^ | 2 (22.2) | 16 (17.8) | - |

Abbreviations: CUP, compassionate use program; pts, patients; cBTKi, covalent bruton tyrosine kinase inhibitor; ECOG PS, Eastern Cooperative Oncology Group performance status; MIPI, Mantle cell lymphoma International. Prognostic Index; sMIPI, simplified MIPI; MIPIc, combined MIPI; CAR-T, chimeric antigen receptor T-cell therapy; BCL-2, B-cell lymphoma-2; PI3K, phosphoinositide 3-kinase.

^a^ This metric is calculated as the percentage of patients who received a prior cBTK inhibitor.

^b^ In cases of discontinuation with multiple reasons, disease progression was prioritized.

^c^ Some of the reasons for discontinuation of treatment included the decision made by the patient, the decision made by the physician, and other miscellaneous reasons.

**Supplemental Table 2.** Restricted mean survival times of patients treated with pirtobrutinib.

|  | DOR | PFS | OS |
| --- | --- | --- | --- |
| Restricted mean, month (95% CI, month) | 9.7 (6.2-NR) | 9.8 (5.9-13.7) | 10.8 (6.8 -14.7) |
| Observation, month | 12.7 | 14.9 | 14.9 |

As the median survival did not fall below 50% in our observation period, we report the restricted mean survival times (RMSTs) using the respective maximum follow-up as the upper limit for DOR, PFS, and OS. In the DOR analysis, only patients showing a response were included.

Abbreviations: RMST, restricted mean survival time; DOR, duration of response; PFS, progression free survival; OS, overall survival; NR, not reached; CI, confidence interval.

**Supplemental Table 3.** Non- and hematologic toxicities of pirtobrutinib documented among patients of the study and approval trial.

| Adverse Event | CUP patients | | BRUIN trial | |
| --- | --- | --- | --- | --- |
|  | TRAE (any grade), No. (%) | TRAE (grade≥3), No. (%) | TRAE (any grade), No. (%) | TRAE (grade≥3), No. (%) |
| **Non-hematologic toxicities** | | | | |
| Hemorrhage | 2 (20) | 0 | 11 (6.7) | 1 (0.6) |
| Elevated liver enzymes | 1 (10) | 0 | 0 | 0 |
| Diarrhea | 1 (10) | 0 | 20 (12.2) | 0 |
| Stomach pain | 1 (10) | 0 | 0 | 0 |
| Dysgeusia | 1 (10) | 0 | 0 | 0 |
| **Hematologic toxicities** | | | | |
| Anemia | - | 0 | 10 (6.1) | 0 |
| Thrombocytopenia | - | 0 | 2 (1.2) | 0 |
| Neutropenia^a^ | - | 0 | 15 (9.1) | 14 (8.5) |

Abbreviations: CUP, compassionate use program; TRAE, treatment-related adverse event.

^a^ Combines neutrophil count decreased, neutropenia, febrile neutropenia, and neutropenic sepsis.
